# Correction Notice: A Study of Safety and Efficacy of Nivolumab and Bendamustine (NB) in Patients With Relapsed/Refractory Hodgkin Lymphoma After Nivolumab Monotherapy Failure

**DOI:** 10.1097/HS9.0000000000000619

**Published:** 2021-07-15

Since the publication of the article entitled “A Study of Safety and Efficacy of Nivolumab and Bendamustine (NB) in Patients With Relapsed/Refractory Hodgkin Lymphoma After Nivolumab Monotherapy Failure” (*HemaSphere.* 2020;4(3):e401), the authors wish to adjust the bendamustine dosing unit of measure in the methods section so as to keep the units consistent throughout the manuscript. The original unit of measure used in the methods section was mg/kg; this is now updated to show as mg/m^2^.

The changes have been made online: https://journals.lww.com/hemasphere/Fulltext/2020/06000/A_Study_of_Safety_and_Efficacy_of_Nivolumab_and.13.aspx

